# Results of Endometrial Sampling in a Tertiary Hospital

**DOI:** 10.7759/cureus.37454

**Published:** 2023-04-11

**Authors:** Alev Esercan, Ismail Demir, Ipek Eskiyoruk, Omer Geckil

**Affiliations:** 1 Obstetrics and Gynecology, Şanlıurfa Education and Research Hospital, Şanlıurfa, TUR

**Keywords:** insufficient material, endometrial polyp, endometrial carcinoma, dilatation and curettage, endometrial sampling

## Abstract

Aim: This study aims to investigate the results of endometrial sampling in a tertiary hospital according to age and symptoms.

Materials and methods: A total of 505 patients who underwent endometrial sampling due to various reasons in our tertiary hospital in the last six months were included in our study, and the pathological results of endometrial sampling were analyzed retrospectively.

Results: The mean age of 505 patients was 45.03 + 7.40 (19-74) years, and 91.5% of the indications were premenopausal abnormal uterine bleeding and 8.5% were postmenopausal bleeding. The most common pathological result of all age groups was proliferative endometrium (37.6%), and the least was the endometrial intraepithelial neoplasia (0.2%). In 9.3% of patients, the result was insufficient material. Of the patients, 88.7% had no additional organic pathology. A total of 30 (5.9%) patients have undergone surgery after endometrial sampling.

Conclusion: It should be kept in mind that despite the pathological result may be insufficient rarely, endometrial sampling should be performed as the first choice in abnormal uterine bleeding.

## Introduction

Abnormal uterine bleeding is the most common complaint in non-pregnant women and affects approximately 10-30% of the population of the reproductive age group [1]. In classical terminology, FIGO (Fédération Internationale de Gynécologie et d'Obstétrique) defines the normal amount of bleeding in menstruation as approximately 37-41 ml, which is expected to be completed in five to seven days. To say that menstrual bleeding is abnormal, there must be 100-130 ml of blood loss or bleeding that results in anemia [2]. With the PALM-COEIN definition defined by FIGO [3], the definition of abnormal uterine bleeding was further elaborated on organic and non-organic causes and divided into definitions. The abbreviation PALM-COEIN represents polyp, adenomyosis, leiomyoma, malignancy and hyperplasia, coagulopathy, ovulatory dysfunction, endometrial, iatrogenic, and not yet classified [3]. As PALM means structural causes, COEIN is for non-structural causes, especially anovulation.

Some methods are available for endometrial sampling, such as dilatation and curettage (D&C) and Pipelle in office standards. Although Clark et al. [4] defined Pipelle as the most commonly used method for endometrial sampling, its disadvantage is that the insufficient material rate is even higher in 13% of patients, especially after menopause. Despite this disadvantage being reduced by the use of D&C [5], one of the most important factors is the experience of the doctor. None of the methods guarantees to obtain adequate specimens.

In our study, we aimed to determine the symptoms of patients with abnormal uterine bleeding, coexisting organic pathologies, pathological reports of endometrial sampling, and the main pathology if the patient underwent a hysterectomy.

## Materials and methods

Three types of patients were included in the study: (i) patients with abnormal uterine bleeding who were over 35 years old; (ii) patients with abnormal bleeding under the age of 35 years whose complaint of irregular uterine bleeding did not regress despite medical treatment; and (iii) postmenopausal patients who applied to the obstetrics outpatient clinic of our hospital due to abnormal uterine bleeding.

A pregnancy test was performed before endometrial sampling and pregnant/aborted patients were not included in the study. Before endometrial sampling, all patients were evaluated with transvaginal ultrasound, and additional findings (cyst, fibroid, intrauterine device, etc.) were recorded on ultrasound. If the patients went to the operation, the pathological final results of the endometrium at the end of the hysterectomy were also recorded. Data for the study were collected retrospectively from the pathology reports of patients who had endometrial sampling in the last six months in Şanlıurfa Training and Research Hospital. In addition, patients' demographic features were collected.

The endometrial sampling procedure was done with a Karman syringe cannula under regional (saddle block) anesthesia. The endometrial samples taken were placed in paraffin blocks, cut with 4-6 µm thicknesses, stained with hematoxylin and eosin (H and E), and evaluated under the microscope.

Ethical approval for this study was obtained from Harran University Ethics Committee, Şanlıurfa, Turkey (dated: 09.01.2023; decision number: 23.01.30). Because the study was designed retrospectively, no written informed consent was obtained from the patients. All procedures were carried out in accordance with the ethical rules and the principles of the Declaration of Helsinki. Statistical analyses were performed using the Statistical Package for the Social Sciences (SPSS version 26.0, IBM Corp., Armonk, NY). The Kolmogorov-Smirnov test was used to verify the normality of the distribution. Mean and median values were used to describe the data characteristics for normal distribution. Categorical data were presented as percentages. The significance level for all tests was defined as p < 0.05.

## Results

A total of 505 patients were included in the study. The mean age of the patients was 45.03 + 7.40 (19-74) years, and 91.5% of the indication for endometrial sampling was for abnormal uterine bleeding and 8.5% was for postmenopausal bleeding.

The most common pathological result for the total age group was proliferative endometrium (37.6%), and the least was endometrial intraepithelial neoplasia (0.2%). In 9.3% of the patients, the result was insufficient material (Table 1).

**Table 1:** Pathology reports of endometrial sampling according to menopausal status AUB: abnormal uterine bleeding; P: polyp; E: endometrial pathology; M: malignancy; FIGO: Fédération Internationale de Gynécologie et d'Obstétrique.

Menopausal status	FIGO classification (PALM-COEIN for AUB)	Premenopausal (n, %)	Postmenopausal (n, %)	Total (n)
Endometrial polyp	AUB-P	75 (15%)	9 (1.8%)	84
Atrophic endometrium	AUB-E	2 (0.4%)	5 (1%)	7
Proliferative endometrium	AUB-E	186 (36.8%)	4 (0.8%)	190
Secretory endometrium	AUB-E	125 (24.7%)	4 (0.8%)	129
Endometrial hyperplasia without atypia	AUB-M	23 (4.5%)	2 (0.4%)	25
Endometrial hyperplasia with atypia	AUB-M	3 (0.6%)	0 (0%)	3
Endometrial intraepithelial neoplasia	AUB-M	1 (0.2%)	0 (0%)	1
Endometrial cancer	AUB-M	0 (0%)	2 (0.4%)	2
Insufficient material		33 (6.5%)	14 (2.8%)	47
Chronic endometritis	AUB-E	14 (2.8%)	3 (0.5%)	17

Of the patients, 88.7% had no additional organic pathology. Of the patients, 3.8% had cervical polyps or ovarian cysts, 0.4% of patients had an intrauterine device for contraception, and 3.4% had myoma uteri.

Thirty (5.9%) patients have undergone surgery after endometrial sampling. A total of 27 out of 30 pathology reports of hysterectomy materials were benign. The other three pathology results were as follows: one each for endometrium cancer, metastasis of cervical cancer, and leiomyosarcoma.

## Discussion

A total of 505 patients were included in the study. Irregular bleeding is usually seen in peri and postmenopausal patients, and the mean age of the patients in our study was 45.03 + 7.40 (19-74) years. That is, our patients were mostly in the perimenopausal period [6]. A total of 43 patients were in the postmenopausal group. The generalized name for abnormal uterine bleeding is dysfunctional uterine bleeding (DUB).

Although there are no organic causes in DUB, abnormal responses occur in the endometrium with abnormalities occurring mostly in the hypothalamic-pituitary-ovarian axis. In our study, the most common pathological finding was proliferative endometrium found in 190 (37.6%) cases. In a study by Vijayaraghavan Sr et al., its occurrence was found as 35% [7]. Secretory endometrium incidence in our study was 25.5%. In the literature, similar incidences of secretory endometrium were found at 18% [7].

In our study, the most common symptom of abnormal uterine bleeding was menorrhagia found in 91.5% of patients. Menorrhagia is the most common complaint in the younger age group and the perimenopausal age group, both in our study and in the literature [7]. A total of 43 patients (8.5%) have undergone endometrial sampling due to postmenopausal bleeding.

Proliferative endometrium rate was the most common pathology because the majority of our patients were in the perimenopausal age group. Because in this age group, endometrial thickening is expected after advanced age, probably associated with obesity and unopposed estrogen. Behera et al. also supported this finding [8].

Atrophic endometrium was found in 1.4% of patients, with the most in the postmenopausal group (1%). In a study by Doraiswami et al. [9], it was found as 2.4%, but the distribution of the number of postmenopausal patients was higher than in our study. While the cause of atrophic endometrium is not known exactly, it is probably due to hypoestrogenism.

Atrophic endometrium was detected in 84 (16.8%) patients, taking the 3rd rank in endometrial pathologies. In our study, in which the proliferation, which is a result of unopposed estrogen, was high, it is also common to see endometrial polyps, which are the result of excess estrogen.

The rate of chronic endometritis in our study was 3.3%, which is similar to the literature (2.5% in Behera et al.'s study [8]). All endometritis were chronic non-specific endometritis and no tuberculosis-related endometritis was found.

In fact, the main purpose of performing endometrial sampling in patients with bleeding disorders is to rule out endometrial cancer. In our study, we captured two postmenopausal patients with endometrial cancer and one perimenopausal patient with endometrial intraepithelial neoplasia. Thirty (5.9%) patients had undergone surgery after endometrial sampling. A total of 27 out of 30 pathology reports of hysterectomy materials were benign. The three pathology results were as follows: one each for endometrium cancer, metastasis of cervical cancer, and leiomyosarcoma. While 30 patients who underwent endometrial sampling were operated on due to abnormal uterine bleeding that did not stop despite medical treatment, one had endometrial cancer, one had cervical cancer metastasis, and one patient had leiomyosarcoma. Endometrial cancer was already diagnosed preoperatively, but the sampling of one patient with cervical cancer metastasis was endometrial hyperplasia with atypia, and the preoperative sampling of the patient with leiomyosarcoma was proliferative endometrium. This showed that there might be an error in the samples of two (0.4%) patients.

Our method for endometrial sampling is a blind examination without ultrasound guidance, and of course, the material taken may be insufficient. Insufficient material was obtained in 6.5% of perimenopausal patients and 2.8% of postmenopausal patients. This may be a limitation of our study and indeed of blind endometrial sampling, perhaps this patient population can be diagnosed by hysteroscopy or ultrasonography-guided endometrial sampling.

Previous studies have tried to find more reliable non-invasive endometrial sampling methods with new endometrial sampling tools such as Endoflower, Tao brush, Li brush, and Endocyte [10]. Tao brush has been described by the Food and Drug Administration [11] as a painless ambulatory endometrial sampling method that prevents contamination of the lower genital tract with endometrial cells. Tao brush is a 3.5 cm brush that is inserted through the cervical canal and rotates around itself three to five times in the uterine fundus to painlessly collect the endometrial cells. In a meta-analysis by Raffone et al. [12], the authors recommended the Tao brush method because of its high sensitivity in detecting premalignant and malignant lesions.

In a study by Wentzensen et al. [13], like cervical cytology, endometrial cytology will be developed through molecular testing by adding extra tests such as human papillomavirus co-testing in cervical cytology.

## Conclusions

As a result, menorrhagia is the most common abnormal uterine bleeding finding, and it is most frequently seen in the perimenopausal age group. Proliferative and secretory endometrium and endometrial polyps are the most common pathologic results. However, in accordance with the purpose of our procedure, although rare, endometrial carcinomas are also being detected. However, it should be kept in mind that the result may be insufficient and a further examination may be required.
